# The potential of federated learning for public health purposes: a qualitative analysis of GDPR compliance, Europe, 2021

**DOI:** 10.2807/1560-7917.ES.2024.29.38.2300695

**Published:** 2024-09-19

**Authors:** Natalie Lieftink, Carolina dos S Ribeiro, Mark Kroon, George B Haringhuizen, Albert Wong, Linda HM van de Burgwal

**Affiliations:** 1 Centre for Infectious Disease Control, National Institute for Public Health and the Environment (RIVM), Bilthoven, The Netherlands; 2 Athena Institute, VU University Amsterdam, Amsterdam, The Netherlands; 3 Centre for Research and Data Services, National Institute for Public Health and the Environment (RIVM), Bilthoven, The Netherlands

**Keywords:** artificial intelligence, data protection, federated learning, GDPR, machine learning, privacy-preserving technologies, public health

## Abstract

**Background:**

The wide application of machine learning (ML) holds great potential to improve public health by supporting data analysis informing policy and practice. Its application, however, is often hampered by data fragmentation across organisations and strict regulation by the General Data Protection Regulation (GDPR). Federated learning (FL), as a decentralised approach to ML, has received considerable interest as a means to overcome the fragmentation of data, but it is yet unclear to which extent this approach complies with the GDPR.

**Aim:**

Our aim was to understand the potential data protection implications of the use of federated learning for public health purposes.

**Methods:**

Building upon semi-structured interviews (n = 14) and a panel discussion (n = 5) with key opinion leaders in Europe, including both FL and GDPR experts, we explored how GDPR principles would apply to the implementation of FL within public health.

**Results:**

Whereas this study found that FL offers substantial benefits such as data minimisation, storage limitation and effective mitigation of many of the privacy risks of sharing personal data, it also identified various challenges. These challenges mostly relate to the increased difficulty of checking data at the source and the limited understanding of potential adverse outcomes of the technology.

**Conclusion:**

Since FL is still in its early phase and under rapid development, it is expected that knowledge on its impracticalities will increase rapidly, potentially addressing remaining challenges. In the meantime, this study reflects on the potential of FL to align with data protection objectives and offers guidance on GDPR compliance.

**Key public health message**

**What did you want to address in this study?**

Machine learning holds great potential to improve public health, yet its use is complicated by the sensitive nature of health data, which are protected under the European Union’s General Data Protection Regulation (GDPR). Federated Learning (FL) is a promising technology that could enhance privacy by keeping data in their original database. In this study we investigated how FL aligns with GDPR principles in the public health sector.

**What have we learnt from this study?**

This study found that FL mitigates many privacy risks associated with sharing personal health data. However, it also highlighted data protection issues, including challenges in verifying data and understanding potential risks. Both GDPR and FL experts faced difficulties assessing FL under the GDPR, indicating significant knowledge gaps. Interdisciplinary approaches are needed to address these challenges and ensure GDPR compliance.

**What are the implications of your findings for public health?**

Our findings can guide public health professionals working with European data on how to use FL while ensuring GDPR compliance. They highlight the need to address specific data protection implications to ensure the safe and effective use of this technology in public health contexts.

## Introduction

The wide application of machine learning (ML) holds great potential to improve public health by supporting data analysis for research and subsequent policy development [1-3]. However, to reap the most benefits from ML techniques, large and diverse datasets are required [4]. Even though there has been a huge increase in the amount of digital data of relevance for public health, its use is often hampered by the inaccessibility of large datasets as they are fragmented across different organisations. Moreover, securing access is strictly regulated by the European Union (EU) General Data Protection Regulation (GDPR), a law that imposes many obligations covering the access, storage, exchange and use of personal data [5,6].

The GDPR protects the privacy of citizens by avoiding unauthorised or unintended disclosure of personal data, which could potentially lead to discrimination or violation of individual rights [7-10]. Consequently, fully anonymous data – those that have been irreversibly altered so that individuals can no longer be identified – are not considered personal data and fall outside the scope of the GDPR [5]. Pseudonymous data, on the other hand, involve the processing of personal data in a way that the data can no longer be attributed to a specific individual without the use of additional information kept separately. While this additional information allows the data provider to re-identify individuals, it has been successfully argued in the European General Court that if a data recipient does not have access to encryption keys that allow re-identification of individuals and has no other lawful means of re-identification, pseudonymous data in the hands of the recipient could also be considered to fall outside the scope of the GDPR [11]. 

**Figure 1:**
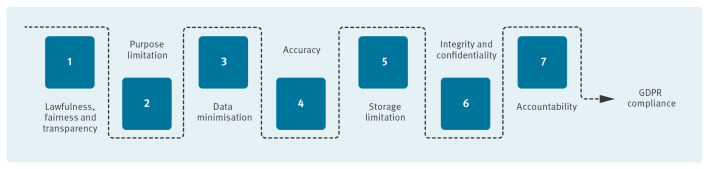
The seven data protection principles that ensure compliance with the European Union’s General Data Protection Regulation

To improve the understanding of the GDPR requirements and to identify measures to ensure compliance, seven data protection principles are laid out in Article 5 (Figure 1) [5]. These principles provide a foundational framework for handling personal data responsibly and ensuring that practices align with GDPR standards.

GDPR: General Data Protection Regulation.

Due to their sensitive nature, the majority of public health data are considered personal data, and therefore difficult to access, share and combine across different institutions, hindering the wide application of ML techniques for data analytics to inform public health policy and practice [6,12]. In order to promote ML-based research requiring large datasets containing personal data, without compromising privacy, the application of federated learning (FL) has received interest as a prospective solution [13-15]. Federated learning is an ML setting that makes it possible to train algorithms collaboratively, and collectively gain insights, without exchanging the data themselves. Instead, FL ensures that the ML process takes place locally in each participating institution, and only model features (e.g. parameters, aggregates or other non-sensitive information) are shared externally from the hub hosting the data. Consequently, the raw data stay at their source [12,16]. Applying this technique could help bridge the gap between the need for protection of privacy and the beneficial use of big data analytics for public health research purposes. However, it is still unknown whether FL is suitable, safe and effective to be widely applied within public health, considering the legal requirements of the GDPR. 

While data protection within ML-based technologies is considered to be of great importance (e.g. to maintain public trust in the technology), studies show that in many technology assessment methods, legal implications are either not given adequate consideration or not taken into account at all [17,18]. This is mostly due to the current lack of ML-specific best practices and guidance [18]. Since public health research uses large amounts of personal (health) data, a responsible way of implementing FL in this field necessitates an assessment of potential data protection issues in light of the GDPR [19]. In this study, we investigated how the GDPR applies to FL when used within the field of public health. The results could be used to inform and raise awareness of public health scientists and policymakers considering or using FL on which data protection aspects they need to address to ensure GDPR compliance [16,20].

## Methods

**Figure 2:**
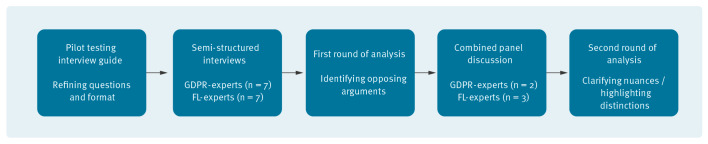
Sequential qualitative research design consisting of two rounds of data collection and analysis, Europe, 2021 (n = 14)

FL: federated learning; GDPR: General Data Protection Regulation.

For this study, we used a sequential qualitative research design consisting of two rounds of data collection and analysis (Figure 2). As expertise in GDPR and FL is often not overlapping, we conducted semi-structured interviews individually with both GDPR and FL experts. After the interviews, the GDPR and FL experts discussed the preliminary findings derived from the analysis in a combined panel discussion. Using these subsequent data collection methods provided the ability to obtain additional in-depth knowledge into the topic, validate the statements across the experts and increase the internal and external validity of the findings [21]. For full clarity and transparency in the research methodology and findings, this study followed the COnsolidated criteria for REporting Qualitative research (COREQ) checklist, which is appended in the Supplement, section 1.

All interviewees were invited to the panel discussion. Five, out of 14 experts, attended with time constraints being the main reason for non-participation.

### Study population and sampling strategy

To qualify for participation in this study, individuals needed to meet either of these criteria: (i) familiarity with designing and implementing FL and/or ML methods for data processing, demonstrated through practical experience (project work) or theoretical expertise (published analysis); or (ii) proficiency in applying GDPR to evaluate compliance from data projects engaged with data analytics. Exclusion criteria comprised not fulfilling these criteria and a lack of proficiency in English or Dutch. Purposive and snowball sampling methods were used as the population was specific and difficult to find [22].

Contact information was retrieved from an Internet search or obtained via the network of the commissioning (research) party, the Dutch National Institute for Public Health and the Environment (RIVM). After inventorying potential participants, they were approached through email and/or LinkedIn. We followed the Chatham House rule of privacy and anonymity during collection and analysis of the data. Before the interview started, the participants received an email containing an information letter and consent form; both are made available in the Supplement, section 2. After agreeing to participate, they received another email containing additional information and a pre-defined set of questions; these can be accessed in the Supplement, section 3.

### Data collection and analysis

To cover all relevant aspects, we used a pilot-tested interview guide based on the seven data protection principles of the GDPR. Participants discussed how these principles would be supported and/or challenged by the application of FL for data analytics in the public health context. The interviews took 45–60 min and were conducted online and recorded via Cisco Webex. New interviews were conducted until data saturation was reached. All interviews were conducted between 13 April and 27 May 2021.

Preliminary findings were presented in a panel discussion to which all experts who participated in the interviews were invited. Five of the 14 experts participated, with the primary reason for non-participation being time constraints. Similarly to the interviews, this panel discussion was both conducted online and recorded via Cisco Webex. Themes in which alignment was found were presented as statements, enabling participants to express agreement or disagreement through poll voting. Themes in which opposing arguments were found were addressed by following up with clarifying questions and group discussions. The panel discussion had a duration of 90 min and was held on 26 May 2021.

All interviews and the panel discussion were transcribed, and a thematic analysis was performed. The data were coded (using Atlas.Ti 9 software) using both inductive and deductive coding strategies [23]. Deductive coding was based on the conceptual framework following from the GDPR principles. Subsequently, we gathered all codes in a coding framework, which is presented in the Supplement, section 4. The entire coding process was performed by a first and second coder (NL and CdSR). Results are presented anonymously.

## Results

### Characteristics of the study population

**Figure 3:**
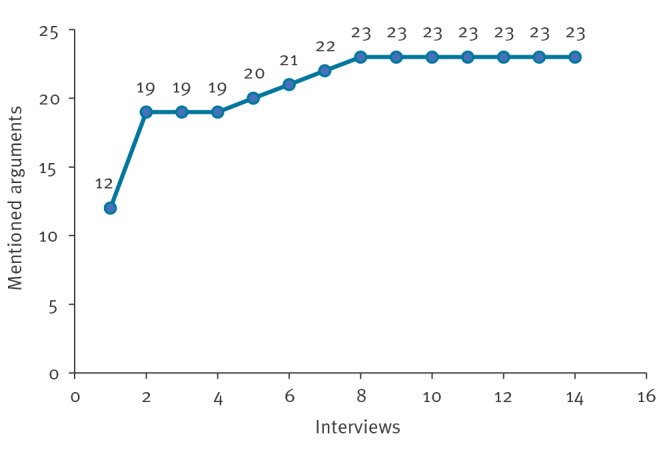
Frequency of arguments mentioned in interviews about the use of federated learning in public health and its alignment with the General Data Protection Regulation, Europe, 2021 (n = 14)

We conducted a total of 14 interviews with key opinion leaders (KOLs). A total of 23 different arguments were mentioned. Data saturation, indicating the number of interviews after which no new arguments were presented in subsequent interviews, was reached after eight interviews (Figure 3). The characteristics of the study population (n = 14) are shown in the Table. The participants consisted of seven FL experts and seven GDPR experts. The majority of experts were based in the Netherlands (n = *7*) and organisationally represented mostly universities (n = 8) and governmental organisations (n = 4). Most experts only had experience in their own area of expertise, however, some also had overlapping knowledge with the other discipline, such as a GDPR expert who focused on assessing ML systems. From all included participants, five attended the panel discussion, consisting of three FL experts and two GDPR experts.

Data saturation was reached after eight interviews. A total of 23 arguments were mentioned.

**Table:** Characteristics of the study population of experts in federated learning and the General Data Protection Regulation, Europe, 2021 (n = 14)

Baseline characteristics	FL experts (n = 7)	GDPR experts (n = 7)	All experts (n = 14)
Geographical representation			
The Netherlands	1	6	7
Switzerland	2	0	2
United Kingdom	2	0	2
United States	1	0	1
Austria	0	1	1
Spain	1	0	1
Organisational representation^a^			
Governmental organisation	3	1	4
Private organisation	0	2	2
University	4	3	7
Non-profit organisation	0	1	1
Level of experience with FL			
No experience	0	6	6
Theoretical experience	3	1	4
Practical experience	4	0	4
Level of experience with the GDPR			
No experience	6	0	6
Theoretical experience	1	0	1
Practical experience	0	7	7

FL: federated learning; GDPR: General Data Protection Regulation.

^a^ Some experts represented more than one organisation.

### Interviews and panel discussion

**Figure 4:**
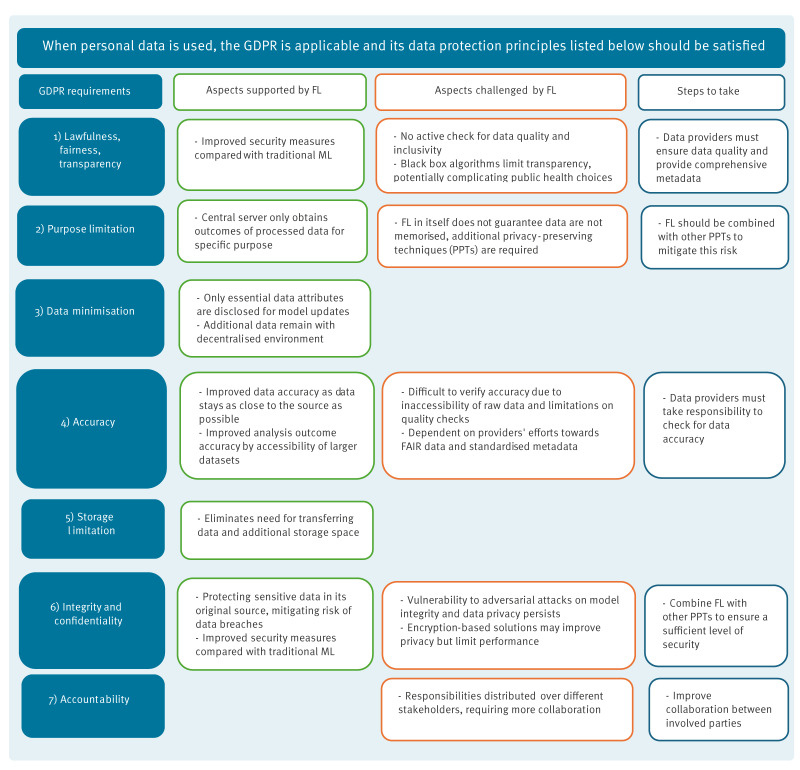
Data protection implications of applying federated learning in advanced analytics in public health, Europe, 2021 (n = 14)

FL: federated learning; GDPR: General Data Protection Regulation; ML: machine learning; PPT: privacy preserving techniques.

The various arguments raised by FL and GDPR experts are further discussed below and visually represented in Figure 4.

### Applicability of the General Data Protection Regulation

In the context of applying FL for processing data in the field of public health, understanding the applicability of the GDPR is essential. Many experts were uncertain about the applicability of the GDPR in the investigated context as they agreed that differentiating between personal, pseudonymised and anonymous data under GDPR standards can be complex and necessitates a case-by-case assessment. This unclarity and challenge in understanding the applicability of GDPR principles becomes particularly evident when applying a novel technology like FL, which is highly technical, with its full impact not fully understood and immediate legal expertise lacking. One GDPR expert explained: *“Even after applying an algorithm, the remaining data is still frequently considered personal data. This is because one must always consider the possibility that outcomes could still be linked back to individuals.”* As the GDPR is not solely concerned with the transfer or sharing of raw data, it could also encompass any information or process that can be used to identify an individual, including that derived from pseudonymous or aggregated data. Due to the sensitive nature of health data, ensuring absolute anonymity when using FL may be very difficult and therefore, complying with the basic principles outlined by the GDPR remains the best course of action.

### Lawfulness, fairness and transparency

The data protection principle of *‘lawfulness, fairness and transparency’* was perceived to be both supported and challenged by the application of FL. Experts noted FL’s potential to support lawfulness because it applies enhanced security measures compared with traditional ML, e.g. reduced centralisation and increased participant control. On the other hand, compliance to fairness was seen as challenging since FL does not allow researchers to actively check data for quality and inclusivity, potentially exacerbating biases. One FL expert mentioned: *“If you apply FL in the context of public health, it is likely that data from a large population is used. This could lead to certain subgroups being underrepresented, resulting in lower quality models or even populations being overlooked during the training process of the model.”* Furthermore, transparency was commonly identified as a hurdle due to the ‘black box’ effect of algorithms, making data processing through FL difficult for humans to comprehend. A lack of transparency could create complications for public health choices that involve FL, since the fundamental rationale behind these decisions remains inexplicable.

### Purpose limitation

Several experts indicated that FL supports the ‘*purpose limitation’* principle as the central server of FL can only obtain the outcomes of the processed data for one specific purpose, which has to be approved by the data hosts. Compared with traditional ML, where the complete dataset is shared and different types of analysis are possible for different purposes, the participant using FL retains more control over what data are processed and for what purpose. However, one FL expert mentioned: *“FL itself doesn't guarantee that it won’t memorise data within the model. Differential privacy is also necessary.”* This expert emphasised that the arguments put forth by the other experts only hold true when FL is combined with other privacy preserving techniques (PPTs).

### Data minimisation

Experts agreed with each other that the principle of ‘*data minimisation*’ is better preserved when using FL than with traditional data processing methods. The FL’s central server has restricted access to data, as only essential attributes are disclosed for model updates. Additional data remain within the decentralised environment, which minimises the exposure of sensitive information.

### Accuracy

The principle of ‘*accuracy’* can be considered both supported and challenged by the application of FL. Some experts argued that FL improves data accuracy, as data stay as closely to the source as possible. This is particularly advantageous in dynamic fields such as public health, where data may change over time as new information becomes available. In addition, the accuracy of outcomes will enhance as FL enables accessibility to a larger number of datasets. However, concerns arose, particularly among GDPR experts, about verifying data accuracy due to FL's inaccessibility to raw data and limitations on quality checks. Hence, compliance with this principle largely relies on data providers' efforts. One FL expert emphasised: *“Operating in an FL environment necessitates grasping metadata, as data itself remains inaccessible. Understanding data creation, biases and interpretation becomes vital. […] This way, you’re able to understand the results correctly and identify potential data pitfalls.”* For this reason, the success of FL projects relies on seamless data interoperability, urging data providers to actively work towards offering findable, accessible, interoperable and reusable (FAIR) data and to engage collaboratively in defining metadata standards.

### Storage limitation

All experts agreed that the *‘storage limitation’* principle is supported by FL, as it eliminates the need for transferring personal data and creating additional storage space. This was argued to be of great importance in public health emergencies, as the ability to access and process data without the burden of extensive storage requirements enables rapid response and decision-making.

### Integrity and confidentiality

Most experts agreed that the data protection principle of ‘*integrity and confidentiality’* is supported by FL because its application allows protection of sensitive data in its original source, simultaneously mitigating the risk of massive data breaches. However, it was acknowledged that FL alone does not guarantee absolute security and privacy as vulnerability to adversarial attacks on model integrity and data privacy persists. To enhance data security and mitigate potential threats, experts recommended combining FL with other PPTs, such as differential privacy and homomorphic encryption [24,25]. One FL expert clarified: *“Solutions exist for data privacy challenges in FL, but they often come with costs, accuracy reduction or limited feasibility in public health due to scale requirements. It's a nuanced trade-off.”* Especially the privacy-performance trade-off was frequently highlighted by various experts. A GDPR expert argued: *“Implementing stringent security measures and encryption might compromise algorithm functionality for specific public health goals.”* Therefore, balancing the levels of privacy and performance demands careful context-specific consideration.

### Accountability

Experts stressed that great care must be taken to acknowledge and comply with the GDPR within an FL project. Processing data through FL was argued to complicate ‘*accountability’*, because responsibilities lie with different stakeholders, e.g. due to the inability to check for data accuracy by data processors. The research process therefore necessitates a more collaborative character requiring a higher level of trust between all parties compared with traditional processing methods.

## Discussion

These expert discussions have shown that applying FL for data analytics to support public health objectives both supports and challenges compliance with the GDPR. Although there has been a predominantly positive discourse surrounding the integration of FL within public health, it is crucial to acknowledge and address the concerns raised by the experts. The challenges posed by the complexity of implementing FL and ensuring the integrity of decentralised data processing are considerable. Despite FL's goal of enhancing privacy by keeping the data localised, it should be noted that they remain susceptible to privacy risks. Ensuring robust security is further complicated by the essential need for transparency and interpretability of outcomes in the public health domain [26]. Consequently, GDPR supervisory boards are urged to specify legal requirements and provide (specific) guidance for ML applications, including FL [27]. Other policies and regulatory frameworks within the EU, such as the Artificial Intelligence (AI) Act, the European Parliament's resolution on automated decision-making processes (12 February 2020) and the European Health Data Space can be supportive in this regard. They aim to ensure trustworthy systems and practices and complement the GDPR, by providing more specific guidance in some practical aspects [28].

While the experts frequently raised similar arguments regarding the discussed topic, certain points were only brought forward by a few experts, such as the persistent risk of data being memorised within the model. These overlooked points could be a result of FL being a relatively new technology, even for experts. This is exacerbated by the lack of defined FL standards and best practices, a circumstance that is likely to change rapidly in the coming years due to increasing interest in the application of FL [29]. Yet, a noticeable knowledge gap persisted between FL and GDPR experts regarding GDPR interpretation. This gap could lead to misconceptions about GDPR’s applicability, hindering assessment of data protection and adequacy for compliance. Such knowledge disparities among stakeholders can hamper innovation development and implementation [30]. A comparable result was found by Wirth and Kolain, indicating legal uncertainty as a key barrier to widespread blockchain technology adoption in the EU market [31]. Similarly, FL’s advancement and integration in public health might be affected due to unclear FL system requirements and legal uncertainty related to practical applications. The observed knowledge gaps emphasise the necessity for interdisciplinary approaches during technology development, design and implementation phases.

The experts highlighted the importance of trust when applying FL for data analytics within public health. Since FL is not primarily designed as a privacy-preserving tool, but instead as a method to avoid raw data transfer and storage, its potential misuse demands vigilance. Furthermore, algorithms are often mistrusted - mostly due to lack of understanding of their functioning - which remains a major barrier to the adoption of ML techniques [32,33].

This general lack of understanding among public health researchers and authorities extends to FL systems, necessitating their reliance on and trust in the expertise of FL developers. Furthermore, the intention behind FL implementation is of great importance, necessitating transparency among implementing actors. Finally, trust among stakeholders involved in the FL process is essential, since responsibility for data quality and data protection lies with different stakeholders. For these reasons, projects involving FL require a collaborative character in order to comply with the GDPR.

Apart from the various levels of trust needed for FL to ensure adequate data protection, its application in public health could also build trust by addressing certain privacy concerns linked to traditional ML. This potential increase in trust might encourage wider data accessibility, benefiting public health considerably. In global crises, such as the COVID-19 pandemic, global data sharing and the use of ML could have accelerated the pace of crucial research [34,35]. However, several issues, including data security, slowed the rate at which data were shared [35]. Federated systems, without necessarily relying on algorithms and ML, were already explored and implemented in the context of COVID-19, as seen in initiatives such as the European COVID-19 Data Portal and digital applications for contact tracing [36,37]. Building on these experiences, in a near future, FL could emerge as a data solution for ML in the field of public health, enabling global sharing and analysis while providing sufficient data protection.

Several strengths and limitations of this study are worth noting. Despite the small sample size of experts, the sequential design and interdisciplinary study population allowed for a comprehensive exploration of a complex subject, which resulted in the identification of experts’ knowledge gaps and increased validity by data triangulation. The focus on the seven data protection principles provided in-depth insight into how the GDPR framework applies to FL on a general level. Other elements relevant to assessing fitness with the GDPR framework, such as various rights for individuals (e.g. the ‘right to be forgotten’, Art. 17(3)(c)) were not explicitly included, but participants were given the opportunity to elaborate on such aspects during interviews and panel discussion [5]. Future research should focus on the elements of the GDPR not included in this study, to gain a more specified understanding of how the GDPR framework applies to FL. The results of the general technology assessment provide initial guidance on compliance with the GDPR but lack specificity. Therefore, research is required to address unsolved challenges, e.g. by further specifying a case study and context to elaborate on concrete steps for action. Interdisciplinary knowledge integration is essential to bridge the identified knowledge gaps and provide proper guidance for successful development and implementation of FL while ensuring sufficient data protection. More knowledge is required on the effects of the shift in responsibilities to ensure data protection and how to establish trust when implementing FL for data analytics within public health.

## Conclusion

The increased attention that FL has received for being a prospective solution to the inaccessibility of public health data in a privacy-focused world has led to many questions. This study provides an overview of data protection principles supported and challenged when applying FL for data analytics within public health, creating initial support for working towards a GDPR-compliant FL system. Future studies should further specify concrete steps for action on a case-by-case approach, enhancing the practical implementation of FL in public health contexts. Since FL is still in its early phase and under rapid development, it is expected that remaining challenges could be addressed by improving the technology and creating more specific legal advice which addresses the particularities of this technology. Furthering the GDPR-compliant application of FL in public health should therefore entail open collaboration and discussion to understand how the remaining data protection challenges could best be addressed.

## Supplementary Data

203000 Supplement
