# Supplementary material for: The potential of federated learning for public health purposes: a qualitative analysis of GDPR compliance, Europe, 2021
Source: Euro Surveill. 2024 Sep 19;29(38):2300695. doi: 10.2807/1560-7917.ES.2024.29.38.2300695 (PMC11484284; doi:10.2807/1560-7917.ES.2024.29.38.2300695)
Supplement: Supplement [file 23-00695_LIEFTINK_Supplement.pdf]

Disclaimer
This supplementary material is hosted by *Eurosurveillance* as supporting information alongside the article 'The Potential of Federated Learning for Public Health purposes: a qualitative analysis on GDPR Compliance, Europe, 2021', on behalf of the authors, who remain responsible for the accuracy and appropriateness of the content. The same standards for ethics, copyright, attributions and permissions as for the article apply. Supplements are not edited by *Eurosurveillance* and the journal is not responsible for the maintenance of any links or email addresses provided therein.

Supplementary Material

These supplements are intended to provide comprehensive support for the research findings presented in the main text. The supplementary material is organized as follows:

- **Supplement S1** – CoreQ Checklist. This includes the checklist used for quality assessment within the study.
- **Supplement S2** – Information letter and consent form. This contains the information letter provided to participants prior to data collection and the consent form used to obtain their informed consent.
- **Supplement S3** – Additional information for participants. This supplement offers further details provided to participants, ensuring they were fully informed about the subjects of the study.
- **Supplement S4** – Coding framework. This document outlines the coding framework used for data analysis, providing transparency into the methods employed.

Supplement S1 - CoreQ Checklist

Table 1. CoreQ (COnsolidated criteria for REporting Qualitative Research) Checklist

Topic	Item No.	Guide Descriptions
**Domain 1: Research team and reflexivity**		
Personal characteristics		
Interviewer/facilitator	1	NL conducted the interviews / GH facilitated the panel discussion.
Credentials	2	MSc (NL) / LLM (GH).
Occupation	3	Researcher and Policy Advisor (NL) / Coordinating Senior Counsel and Public Health Lawyer (GH).
Gender	4	Female (NL) / Male (GH).
Experience and training	5	Both experienced in qualitative academic research.
Relationship with participants		
Relationship established	6	NL and GH had no prior relationship with the participants.
Participant knowledge of the interviewer	7	One GDPR-expert worked with the facilitator of the panel discussion (GH) at the RIVM. One FL-expert worked at the RIVM as well, but was not directly involved with the interviewer and/or facilitator. All participants were informed by email about the research purpose and the researchers' goals.
Interviewer characteristics	8	Both researchers' roles were to conduct and support the interviews, ensuring all topics were covered and no bias was introduced. This was reported at the start of the interview and panel discussion.
**Domain 2: Study design**		
Theoretical framework		
Methodological orientation and theory	9	The study was guided by a thematic framework analysis.
Participant selection		
Sampling method	10	Snowball- and purposive sampling were used to select the participants.
Method of approach	11	Participants were recruited through RIVM contacts and researchers in the field. They were approached through email and/or LinkedIn.
Sample size	12	This study included 14 individual participants, of which 5 participated in both the interview and the panel discussion.
Non-participation	13	Although all 14 interviewees were invited to the panel discussion, only 5 participated. Participants primarily declined due to time constraints.
Setting		
Setting of data collection	14	Interviews and the panel discussion were conducted online due to COVID-19 restrictions.
Presence of non-participants	15	Only the researchers and participants were present during the data collection sessions.
Description of sample	16	Participants included GDPR and Federated Learning (FL) experts. Demographics of the participants are provided in Table 1.
Data collection		
Interview guide	17	A pilot-tested interview guide based on GDPR principles was used. Researchers were allowed to use prompt questions during the interview.
Repeat interviews	18	No repeat interviews were carried out. However, a panel discussion was conducted to discuss and verify the preliminary findings from the interviews.
Audio/visual recording	19	All interviews and the focus group were recorded via Cisco Webex.
Field notes	20	Notes were made by a researcher during both the interviews and the panel discussion.
Duration	21	Interviews lasted approximately 45-60 minutes; the focus group lasted 1.5 hours.
Data saturation	22	Data saturation was reached after 8 of the 14 interviews.
Transcripts returned	23	A member-check was conducted at the end of each interview. Additionally, the preliminary findings from the interviews were discussed and verified during the panel discussion.
**Domain 3: Analysis and findings**		
Data analysis		
Number of data coders	24	The data was coded by a first and second coder.

Description of the coding tree	25	The coding process involved both inductive and deductive strategies.
Derivation of themes	26	Themes were derived through thematic analysis after analyzing the data.
Software	27	Atlas.Ti 9 was used for coding.
Participant checking	28	Preliminary findings were discussed in a panel discussion with participants.
Reporting		
Quotations presented	29	Direct quotes from participants were used to support findings. Quotations were identified by participant numbers.
Data and findings consistent	30	Findings were aligned with the data collected, validated through group discussions and poll voting within the panel discussion.
Clarity of major themes	31	Major themes and sub-themes were clearly presented.
Clarity of minor themes	32	Minor themes were also addressed in the analysis.
Additional considerations		
Ethical Considerations	33	The study adhered to the Chatham House rule for privacy and anonymity, and Dutch law (WMO) did not require ethics approval due to the nature of the study.
Consent	34	Participants received an information letter and provided verbal informed consent at the beginning of the interviews / panel discussion.

Supplement S2 - Information letter and Consent Form

Introduction
You are invited to take part in a research project investigating data protection issues of applying Federated Learning (FL) in the field of public health. Your participation is completely voluntary. This form explains the main goals of the project and the research design detailing the data collection, processing/analysis, and publication processes. This information is provided to guarantee informed decision-making by the participants. Any questions and doubts regarding this document can be clarified in the introduction of the interview appointment.

Purpose of the study
This research is commissioned by the RIVM, as the Netherlands Institute for Public Health and the Environment, under the two projects of the EU-H2020 VEO project and the RIVM FL-project.

About the VEO project
The EU-H2020 VEO project aims at establishing an observatory for early warning, risk assessment and monitoring of emerging infectious diseases and antimicrobial resistance. In this project, the RIVM is responsible for the investigation of Ethical, Legal and Social Implications (ELSI) of data sharing to support infectious disease management. More information of this project is available at: https://www.rivm.nl/en/international-projects/veo.

About the RIVM-FL project
The RIVM FL-project aims to gain knowledge on FL as a method to learn from distributed data processing as a potential tool for data sharing, and to understand under what circumstances this approach is a solution to barriers in data sharing, especially when organizations are concerned with GDPR restrictions and compliance.

About our study
We aim to investigate which are the challenges and opportunities for applying FL in the context of public health for RIVM and its partner institutes/projects. This research will support public health professionals to understand the data protection issues of sharing and processing data using FL in order to improve public health research and outcomes, by providing insights in the data protection aspects that should be taken into account before, during and after implementing FL. The main reason for conducting this study is to help answering the following research question: *'What potential data protection issues arise when using Federated Learning in the field of public health?'.*

Description of the study
Participation in this study is through a semi-structured interview, informed by a pre-defined interview guide that will be shared with the participants before the interview takes place. Both FL- and GDPR-experts will be interviewed to provide a clear overview of all relevant data protection issues. The interviews will take place via Cisco Webex, due to the current Covid-19 pandemic, and will be conducted by two researchers. Other researchers from the team might join the interviews when convenient. The interview will be audio recorded and transcribed verbatim. A check for understanding and interpretation of your responses will be provided by the end of the interview. You can also request to receive your transcripts after the interview. We will send you the final report once finished for final check before publication.

Withdrawal from the study
Your participation in the study is completely voluntary and you may choose to stop participation and withdraw the data provided at any time without having to give a reason for this. Your decision to stop participating, or to refuse to answer particular questions, will not have negative consequences. In the event you withdraw from the study, all associated data collected will be immediately destroyed wherever possible.

Confidentiality and data protection
In the interview, the collection of sensitive information will be avoided as much as possible, restricting to the information relevant to the research objectives. The interview will be recorded in order to analyse it afterwards thoroughly. During this process, the data will be stored in a safe map only the research

team will have access to. The recording of the interview will be transcribed to an anonymized text and will be immediately deleted afterwards. This means all information supplied for the research will be held in confidence and no information present in the report or publication of this research will be linkable to individuals or organizations. The anonymised data will be stored at the RIVM for 10 years according to the FAIR principles. Confidentiality will be provided to the fullest extent possible by law, overseen by our data protection officer. The collected information will be used for scientific purposes and only for the goals hereby mentioned and will result in a report for the EU-H2020 VEO-project, the RIVM FL-project, the Vrije Universiteit Amsterdam and may be published as a scientific article.

After careful assessment, we kindly ask you to reply to the interview invitation email giving your consent to participate in our study and have your data managed according to the information hereby provided. If you have any questions regarding the study or the data processing, feel free to contact our team and/or clarify those in the beginning of the interview appointment.

Supplement S3 - Additional Information for Participants

Introduction

In this document, additional information is provided that will help you answering the questions during the interview. You will find more information about Federated Learning and the General Data Protection Regulation (GDPR) and its principles. In the final part of this document, a pre-defined set of questions is presented, in order for you to get acquainted with these before the interview starts.

Introduction to Federated Learning (FL)

Privacy Preserving Techniques (PPT) are valuable tools to protect privacy when sharing data for research purposes, helping to overcome barriers in data sharing. Federated Learning (FL) is a type of PPT where the actual data to be used in the analysis does not need to be shared, but stay on the original data repository, while the research question and analysis tool (in the form of machine learning algorithms) travel to the data repositories, process the data and share only the analysis results (e.g., parameters, aggregates or other non-sensitive information) with the outside researchers. This approach stands in contrast to traditional centralized machine learning techniques where all the local datasets are uploaded to one server.

The model is trained in a distributed way: the various databases locally compute encrypted training gradients, and send masked results to the federated server to learn a global model, the federated server securely aggregates the results, the server send back the model updates and the databases locally update their models.

As a new technology, a machine learning setting, FL has a lot of potential to be applied to support data sharing for public health research and action. However, a responsible way of doing it is only possible after the assessment of possible data protection issues in light of the GDPR is performed, since public health research uses large amounts of personal (health) data. Therefore, our study aims to investigate the data protection issues of applying FL in the field of public health.

Introduction to the GDPR

The GDPR is a law that imposes strict obligations regarding the storage and exchange of personal data, in order to protect privacy. It states that personal data includes *'any information relating to an identified or identifiable natural person ('data subject'); an identifiable natural person is one who can be identified, directly or indirectly'* (EU, 2018). Because of the sensitive nature of the majority of public health data, it is extremely difficult to share and combine datasets across different institutions, simultaneously hindering the wide application of AI techniques in the field of public health.

To go a bit more in depth into the GDPR and the specificities for complying with this legal framework, 7 data protection principles were identified. In Table 1, an overview of the principles, their definitions, and operationalisations is presented.

Table 1. The principles, their definitions and operationalisations.

Principle	Definition	Operationalisation
**1) Lawfulness, 2) fairness and 3) transparency**	"Personal data shall be processed while 1) an appropriate lawful basis (or bases) is identified; 2) the effect it has on the individuals concerned is considered and any adverse impact could be justified; 3) the transparency obligations of the right to be informed are complied with by being open and honest at all times."	1) Examples of legal bases are: consent, contract, legal obligation, vital interests, public task or legitimate interests;<br>2) The effect of the application of FL on the data subjects;<br>3) Transparency means full information is provided to the data subjects (for instance the retention periods and the range of their individual rights).
**Purpose limitation**	"Personal data shall be collected for specified, explicit and legitimate purposes and not further processed in a manner that is incompatible with those purposes."	Public health institutes and researchers should be able to define a purpose before the processing of personal data using FL. No further processing beyond the predefined use is allowed, unless further processing is needed for exceptional purposes, such as the public interest or scientific research.

**Data minimization**	''Personal data shall be adequate, relevant and limited to what is necessary in relation to the purposes for which they are processed."	Public health institutes and researchers should ensure all personal data used is limited to what is necessary.
**Accuracy**	''Personal data shall be accurate and, where necessary, kept up to date."	The personal data used in FL have to be up to date. When data is inaccurate, it must be deleted or corrected. This principle is closely linked to individual's *right to rectification*, which gives individuals the right to have inaccurate personal data corrected.
**Storage limitation**	''Personal data shall be kept in a form which permits identification of data subjects for no longer than is necessary for the purposes for which the personal data are processed."	Public health institutes and researchers should limit the storage of personal data while using FL in the field of public health.
**Integrity and confidentiality**	''Personal data shall be processed in a manner that ensures appropriate security of the personal data, including protection against unauthorised or unlawful processing and against accidental loss, destruction or damage, using appropriate technical or organisational measures."	Public health institutes and researchers should ensure the personal data is appropriately secured, for instance using differential privacy, in order to ensure the principle of integrity and confidentiality is met when using FL.
**Accountability**	''The data controller shall be responsible for, and be able to demonstrate compliance with the GDPR."	Public health institutes and researchers are responsible for complying with the GDPR. Moreover, they have to be able to show how this compliance is met.

Pre-defined set of questions for FL-experts

1. From your knowledge and experience, what are the most important solutions Federated Learning provides for data sharing and processing?
2. From your knowledge and experience, what are the most important challenges regarding data protection related to the application of FL in the context of public health?
3. Which of these data protection principles laid out in the GDPR do you think are underserved by or challenge the application of Federated Learning with regard to compliance?
4. How do you think these underserved issues could be best addressed?

Pre-defined set of questions for GDPR-experts

1. From your knowledge and experience, do you think the application of FL in the field of public health is an improvement to data protection and could contribute to compliance with the GDPR?
2. From your knowledge and experience, do you think the application of FL in the field of public health still have to implement legal mechanisms for GDPR compliance?
3. Which of these 7 data protection principles do you think are underserved by or challenge the application of Federated Learning with regard to GDPR compliance?
4. How do you think these underserved issues could be best addressed?

Supplement S4 - Coding Framework

Code theme	Codes	Example phrases	Support by FL-experts	Support by GDPR-experts
**Applicability of the GDPR**	Anonymity is not fully guaranteed by the use of FL; challenges exist in differentiating between personal and anonymous data	"The more you aggregate, the greater the likelihood that the data will truly be anonymous; however, achieving this is quite challenging. From a legal perspective, I would always advise assuming that you are still processing personal data." (GDPR2)	FL1, FL2, FL4, FL5	GDPR2, GDPR4, GDPR5, GDPR6, GDPR7
	Challenges in defining privacy	"From this we can get the idea that privacy is not tangible, right. It is not quantifiable, it's something we're afraid of. It's just a gut feeling, is it private? I don't think the answer to this question will never be yes." (FL1)<br><br>"Even after applying an algorithm, the remaining data is still frequently considered personal data. This is because one must always consider the possibility that outcomes could still be linked back to individuals." (GDPR2)<br><br>"In terms of privacy, there is a large gap between theory [...] and also, what is the exact privacy definition people care about?" (FL2)	FL1, FL2, FL4, FL5	GDPR2
**Lawfulness, fairness and transparency**	Improved data security in FL	"This is a very effective security measure because the original data is not actually shared." (GDPR1)<br><br>"It is definitely a big step forward in preserving privacy, compared to the traditional way of collecting all of the data in a central place, and often stay there with all the risks involved." (FL4)	FL1, FL2, FL3 FL4	GDPR1, GDPR2, GDPR3
	Limited data quality and inclusivity evaluation in FL	"If you apply FL in the context of public health, it is likely that data from a large population is used. This could lead to certain subgroups being underrepresented, resulting in lower quality models or even populations being overlooked during the	FL2, FL4, FL5	GDPR1

		training process of the model." (FL2)		
	Lack of transparency due to the "black-box" effect of algorithms	"Transparency requires particular attention in this situation, as it involves a black box analysis, and it is important to be able to explain to the data subjects exactly what has happened with their data." (GDPR2)	FL2, FL3, FL7	GDPR1, GDPR2, GDPR4, GDPR6
**Purpose limitation**	FL allows data to be processed for one specific purpose	"The model is created for a given purpose. In a decentralized context like FL, acquiring additional data is necessary to train the model for alternate purposes. However, in the traditional centralized context, as a data subject, there is little you can do if your data is processed for additional purposes." (FL3)	FL2, FL3, FL5	GDPR2, GDPR7
	The "client" retains control over what data is processed and for what purpose when using FL	"As a client, I have much more control over what happens to my data, when not all my data is stored in one central server." (FL3)	FL1, FL3, FL5, FL6	GDPR5, GDPR6
	FL models could memorize training examples	"FL itself doesn't guarantee that it won't memorize data within the model. Differential privacy is also necessary." (FL6)	FL1, FL2, FL5, FL6	GDPR1
**Data minimization**	Less access to data when using FL compared to traditional ML	"Data minimization, that is clear, that's probably a big asset of FL, because the data is extremely limited on the central server." (FL3)	FL1, FL2, FL3, FL4, FL5	GDPR1, GDPR2
**Accuracy**	Data stays close to the source in FL which enhances data accuracy	"Using data at the data source keeps it as close as possible being accurate, because copying data normally makes it less accurate." (FL4)	FL4	GDPR7
	Inability to check data quality	"I have obligations towards the data subjects to ensure that the correct data is used; however, I have no visibility into the data to verify its accuracy or whether it is up to date." (GDPR1)<br><br>"It's actually one of the challenges of FL, right? The issue lies in whether the data provided by clients is accurate and relevant to the training process." (FL1)	FL1, FL2, FL4, FL5	GDPR1, GDPR2, GDPR6
	Need for meta-data, FAIR principles and clear agreements among participating clients to ensure accuracy	"Operating in an FL environment necessitates grasping metadata, as data itself remains inaccessible. Understanding data creation, biases, and interpretation	FL4	GDPR1, GDPR2, GDPR4

		becomes vital. […] This way, you're able to understand the results correctly and identify potential data pitfalls." (FL4)		
**Storage limitation**	No need for extra storage with FL	"Besides promoting data minimization, I also believe it reduces security risks since less personal data is stored across various locations." (GDPR2)<br><br>"From a systemic standpoint, FL allows for the utilization of data on a large scale without the need for data storage." (FL1)	FL1, FL2, FL4, FL5	GDPR1, GDPR2, GDPR5, GDPR6, GDPR7
**Integrity & confidentiality**	FL allows protection of sensitive data in its original source	"FL really helps in preserving the integrity and confidentiality principle, since it is a very good security measure." (GDPR1)	FL1, FL3, FL4, FL7	GDPR1, GDPR2, GDPR5, GDPR6, GDPR7
	Absolute security and privacy are not guaranteed (potential for attacks)	"FL helps, but it is not an absolute guarantee for privacy, even if differential privacy is used. Unless an epsilon budget is used to achieve exactly no risks, but this approach will lead to hardcore reduction in utility." (FL6)	FL1, FL2, FL3. FL5, FL6	-
	Demand for performance and accuracy can challenge integrity and confidentiality	"Solutions exist for data privacy challenges in FL, but they often come with costs, accuracy reduction, or limited feasibility in public health due to scale requirements. It's a nuanced trade-off." (FL1)<br><br>"Implementing stringent security measures and encryption might compromise algorithm functionality for specific public health goals." (GDPR6)	FL1, FL2, FL3, FL5, FL6	GDPR3, GDPR6
**Accountability**	Responsibilities lie with different stakeholders, requiring trust	"If I hold data for a certain specific set of people, I'm still responsible for that data and making sure it's safe, GDPR-compliant, etc. But if you have an environment with 10 hospitals that together do FL on 10 different data sources, you don't have one organization that is responsible or accountable for when something goes wrong. [...] There need to be a level of trust involved between all of these organizations." (FL4)	FL1, FL2, FL3, FL4, FL5, FL7	GDPR1, GDPR2, GDPR4, GDPR5, GDPR6, GDPR7
	Need for more guidance in the GDPR on the use of FL	"I think one of the problems with the GDPR at the moment is that every company is implementing it	FL1, FL4, FL5, FL6	GDPR2, GDPR3

		in their own way. [...] There is no single way to easily check whether companies are compliant. I think privacy and technologies can really improve on this kind of burden that the GDPR brings in terms of verification.” (FL5)		
